# Bilateral blindness secondary to optic nerve ischemia from severe amlodipine overdose: a case report

**DOI:** 10.1186/s13256-017-1374-4

**Published:** 2017-08-03

**Authors:** Raymond Kao, Yves Landry, Genevieve Chick, Andrew Leung

**Affiliations:** 1 Schulich School of Medicine & Dentistry, Western University, London, ON Canada; 2 Department of Radiology, London Health Sciences Centre, University of Western Ontario, London, ON Canada; 3 Division of Critical Care, Department of Medicine, London Health Sciences Centre, Western University, London, ON Canada

**Keywords:** Calcium channel blocker, Optic atrophy, Cortical blindness

## Abstract

**Background:**

Calcium channel blockers are commonly prescribed medications; calcium channel blocker overdose is becoming increasingly prevalent. The typical presentation of a calcium channel blocker overdose is hypotension and decreased level of consciousness. We describe a case of a calcium channel blocker overdose that led to bilateral cortical blindness, a presentation that has not previously been reported.

**Case presentation:**

A 49-year-old white woman with known bilateral early optic atrophy presented to our hospital with hypotension and obtundation following a known ingestion of 150 mg of amlodipine. She was transferred to our intensive care unit where she was intubated, mechanically ventilated, and required maximal vasopressor support (norepinephrine 40 mcg/minute, epinephrine 40 mcg/minute, and vasopressin 2.4 units/hour) along with intravenously administered crystalloid boluses. Despite these measures, she continued to deteriorate with persistent hypotension and tachycardia, as well as anuria.

Intralipid emulsion therapy was subsequently administered to which no initial response was observed. A chest X-ray revealed diffuse pulmonary edema; intravenous diuresis as well as continuous renal replacement therapy was initiated.

Following the initiation of continuous renal replacement therapy, her oxygen requirements as well as urine output began to improve, and 3 days later she was liberated from mechanical ventilation. Following extubation, she complained of new onset visual impairment, specifically seeing only red-green colors, but no objects. An ophthalmologic examination revealed that this was due to bilateral optic atrophy from prolonged hypotension during the first 24 hours after the overdose.

**Conclusion:**

Persistent hypotension in the setting of a calcium channel blocker overdose can lead to worsening optic atrophy resulting in bilateral cortical blindness.

## Background

Calcium channel blockers (CCBs) are commonly prescribed cardiovascular medications, used in the treatment of hypertension, angina pectoris, cardiac arrhythmias, migraine headaches, and some circulatory conditions such as Raynaud’s syndrome. CCB toxicity, caused by intentional or unintentional overdose, has devastating systemic effects. According to the 2002 annual report of the American Association of Poison Control Centers Toxic Exposure Surveillance System, 16% of all cardiovascular drug exposures were due to CCBs, however, this class of drugs accounted for 38% of deaths [1].

CCB overdose is known to be associated with hypotension and decreased level of consciousness. A case report from 1992 describes a female who developed a stroke, a rare complication, after a verapamil overdose [2]. To the best of our knowledge, this is the first reported case of bilateral cortical blindness following a severe CCB overdose.

## Case presentation

A 49-year-old white woman was brought to our emergency department (ED) after an intentional overdose with 150 mg of amlodipine, 60 mg of escitalopram, and 6 mg of risperidone. Her past medical history included hepatitis C from a blood transfusion in 1993, remote intravenous drug use, diverticulitis, hypertension, mild chronic obstructive pulmonary disease, gastroesophageal reflux disease, anxiety, and depression. She was also seen by the ophthalmology service in 2007 with regards to declining visual acuity, secondary to early optic atrophy.

She was seen by family to be normal at 11:00 on the day of admission to our hospital, and was subsequently found by family members to have an altered level of consciousness at 13:50, at which point the emergency services were called.

Upon arrival to our ED at 14:00, she was found to be hypotensive with a blood pressure of 84/38 mmHg, tachycardia with a heart rate of 117 beats/minute, and obtunded with a Glasgow Coma Scale (GCS) of 9/15. She was immediately intubated for airway protection, placed on a cardiac monitor, and an arterial and central venous catheter was inserted. The initial resuscitation consisted of a bolus 2 liters of crystalloid administered intravenously, 2 g of calcium gluconate administered intravenously, 5 mg of glucagon administered intravenously, 1 ampule of dextrose 50% in water (D50W) administered intravenously followed by 70 units of bolus short-acting insulin administered intravenously followed by 70 units/hour infusion. She was also given 50 g of activated charcoal via a nasogastric tube for gastric decontamination. The Critical Care team was notified, and she arrived in our Intensive Care Unit at 14:30. Upon arrival, she required 30 mcg/minute of norepinephrine, 30 mcg/minute of epinephrine, and 2.4 units/hour of vasopressin to maintain a mean arterial pressure greater than 65 mmHg.

**Fig. 1:**
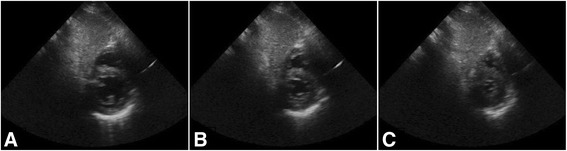
Point-of-care transthoracic echocardiogram subcostal short-axis views. **a** Short-axis view of the *left* ventricle at the level of the papillary muscles reveals the *left* ventricle initially in diastole, **b** followed by mid-systole, and **c** in late systole. Symmetric contraction of the *left* ventricle is appreciated until near obliteration of the *left* ventricle cavity in late systole, which is indicative of a hyperdynamic and under-filled *left* ventricle

A point-of-care transthoracic echocardiogram was performed at 17:00 to evaluate her profound shock, which demonstrated an under-filled hyperdynamic left ventricle (Fig. 1). There was no right ventricle (RV) dilatation, and RV systolic function was intact. This finding was indicative of a severe vasodilatory state causing her shock, as opposed to cardiogenic shock. We began aggressive administration of intravenously administered boluses with crystalloid fluids infused through a Level 1 rapid infuser. Initially, she improved hemodynamically with each 1 L bolus, demonstrating that she was preload-dependent in the setting of her vasoplegia. Her catecholamine requirements decreased significantly, norepinephrine 40 mcg/minute and epinephrine 40 mcg/minute to 25 mcg/minute and 10 mcg/minute, respectively, after a total of 23 liters of intravenously administered crystalloid boluses were given. She was maintained on the vasopressin 2.4 units/hour throughout the resuscitation process.

**Fig. 2:**
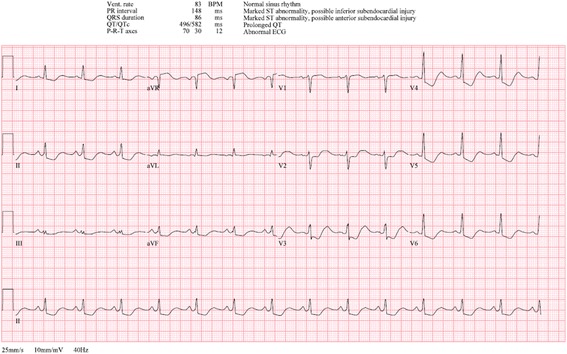
A 12-lead electrocardiogram demonstrating features of hypokalemia including increased amplitude and width of the P-waves, T-wave flattening and inversion, ST depression, and prominent U wave formation best seen in the precordial leads. Apparent QTc prolongation is observed due to fusion of the T and U waves. *BPM* breaths per minute, *ECG* electrocardiogram

Despite continuous repletion of potassium with intravenously administered potassium chloride and orally administered potassium chloride, she developed refractory hypokalemia with associated changes on the electrocardiogram (ECG; Fig. 2). She also required intravenously administered dextrose boluses despite a 10% dextrose infusion. Given the hypokalemia and hypoglycemia that were becoming problematic to treat, the decision was made to abandon hyperinsulinemic-euglycemia therapy in this patient, as she did not appear to have a strong component of insulin resistance.

**Fig. 3:**
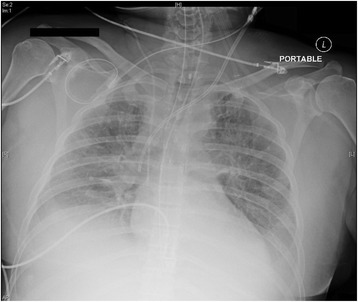
Portable anterior-posterior chest X-ray revealing diffuse bilateral opacities in keeping with pulmonary edema

She was mechanically ventilated on assist-control ventilation with a respiratory rate of 18 breaths/minute, pressure control ventilation at 19 cmH_2_O, positive end-expiratory pressure (PEEP) 5 cmH_2_0, and fraction of inspired oxygen (FiO_2_) 40% with oxygen saturation of 94%. Following 23 liters of fluid resuscitation, her oxygenation requirements greatly increased due to the volume overload causing significant pulmonary edema. Her mechanical ventilation requirements increased to assist-control ventilation rate of 27 breaths/minute, pressure control 36 cmH_2_O, PEEP 14 cmH_2_0, and FiO_2_ 100% to maintain oxygen saturation in the mid 80%. A portable chest radiograph confirmed that she had diffuse pulmonary edema (Fig. 3) and she remained anuric. At 22:15, the nephrology service was consulted to initiate continuous renal replacement therapy (CRRT) for volume overload. At approximately 22:35, we discontinued administration of intravenously administered fluid boluses, minimized all intravenously administered fluid input, and administered a 200 mg bolus of intravenously administered furosemide. She had a good response and her urine output increased to 100 to 225 mL/hour. At 22:50 she continued to deteriorate with low blood pressure, tachycardia, and we proceeded to administer to her 1.5 mL/kg of 20% lipid emulsion therapy, to which she did not initially respond.

The cardiovascular surgery service was also consulted to assess the potential need for veno/venous extracorporeal membrane oxygenation (ECMO). At 23:30, the CRRT was started and she responded very well and ultimately did not require ECMO. At 00:30, her oxygenation status began to improve, with oxygenation saturation >90% on 100% FiO_2_. From 00:30 to 10:30, she had gradual and complete reversal of her shock state. She was slowly weaned off all vasopressors, and continued to make urine after an initial 8 hours of complete anuria postadmission. By 13:00 the following day, she was off CRRT, with a urine output up to 500 ml/hour, thought to be secondary to post-acute tubular necrosis (ATN) diuresis. Her oxygen requirements continued to decrease and by 10:15 she was requiring 60% FiO_2_ to maintain an oxygen saturation of 96 to 98%.

**Fig. 4:**
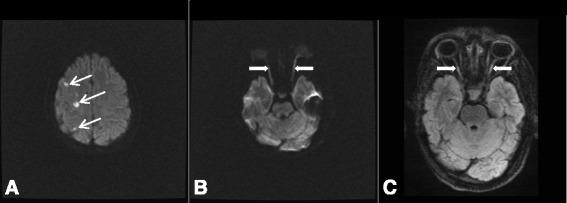
Magnetic resonance imaging of patient’s brain. **a** Axial diffusion-weighted image demonstrating small strokes to the *right* frontal and parietal lobes (*white arrows*). **b** and **c** are axial diffusion-weighted image and fluid-attenuated inversion recovery image demonstrating bilateral optic nerves thickening and increased signal compatible with ischemic injury (*white arrows*)

On clinical examination, she continued to recover and demonstrated no persisting organ dysfunction. Three days after admission, she was extubated and liberated from mechanical ventilation. She was alert and oriented, but complained of new onset visual impairment, specifically only seeing red and green colors, but unable to see any objects. Of importance, her visual acuity was relatively intact prior to admission with the exception of the stable optic atrophy. The remaining neurologic examination was otherwise normal. A magnetic resonance imaging (MRI) of her brain showed a few small foci of high signal on the diffusion-weighted image (DWI) sequence in her right frontal and parietal lobes consistent with acute infarcts (Fig. 4a). These may have been from emboli to the right middle cerebral artery (MCA) territory. Alternatively, these may be deep watershed infarcts between the anterior cerebral artery (ACA) and MCA territories. In addition, both optic nerves were mildly thickened with high signal on DWI and fluid-attenuated inversion recovery (FLAIR), consistent with cytotoxic edema from infarction (Fig. 4b, c). A lumbar puncture was performed which showed an elevated opening pressure (18 mmHg), but otherwise normal cerebrospinal fluid, and no evidence of central nervous system (CNS) vasculitis. Subsequent ophthalmologic evaluation revealed she had no light perception bilaterally despite best corrected vision, no light response, and mid-dilated pupils bilaterally. The intraocular pressure of her right and left eyes was 14 mmHg and 16 mmHg, respectively. Examination of the anterior segment was unremarkable bilaterally. Posterior segment examination revealed no evidence of disc edema, hemorrhage, retinitis, or vasculitis bilaterally.

Both ophthalmology and neuroradiology concluded that her blindness was probably due to optic nerve injury from the prolonged hypotension during the first 24 hours after the overdose. She was discharged from hospital 8 days postadmission in a stable condition; however, she was still burdened by binocular blindness. She was subsequently seen by ophthalmology as an out-patient; an examination again revealed no light perception in both eyes and complete nonreactivity to both pupils, consistent with complete optic atrophy suggesting severe optic nerve damage.

## Discussion

Cardiovascular medications were the seventh most common class of drug exposure reported to poison control centers of the USA in 2014 [3]. The 2014 annual report of the American Association of Poison Control Centers’ National Poison Data System identified 3.96% of cases with multi-substance exposure and 2.36% due to single substance exposures. Cardiovascular medication overdose accounted for the second and third highest number of fatalities in multi-substance and single substance exposures, respectively [3]. Of importance, this drug class had the fourth greatest increase in serious exposures per year from 2000 to 2014 [3]. CCB overdose has been proportionally less common than other cardiovascular medications, such as beta-blockers. In 2002, CCBs accounted for only 16% of cardiovascular medication exposures, while causing 38% of fatalities of this drug class [1]. This emphasizes the lethality of CCB overdose, as well as the importance of discussing individual cases to better understand the physiology and individual drug effects on a case-by-case basis.

CCBs are used in a variety of clinical settings to treat hypertension, angina pectoris, Raynaud’s syndrome, migraine headaches, and tachyarrhythmias [4]. CCBs can be subdivided into two major categories based on their predominant physiologic effects. The dihydropyridines (amlodipine, nifedipine, felodipine, nimodipine) preferentially block the L-type calcium channels in the peripheral vasculature. This results in vasodilatory effects without significant effect on cardiac contractility or chronotropy. The nondihydropyridines (verapamil, diltiazem) inhibit the transmembrane influx of calcium into myocardial fibers and pacemaker cells, with relatively little effect on vascular smooth muscle cells [5]. Amlodipine is structurally related to nifedipine, although it differs because of the presence of a basic amino side chain in the 2 position of the dihydropyridine ring [5]. Of interest, this amino group is positively ionized at physiologic pH, which may account for the unique properties of this CCB. In general, CCBs are characterized by high rates of clearance, low volumes of distribution, and short half-lives. On the other hand, pharmacokinetic studies of amlodipine have revealed it to possess a high volume of distribution and a low rate of elimination [5]. In addition, amlodipine is highly protein-bound and metabolized primarily by the liver [4]. There is also a dose-dependent change in pharmacokinetic clearance from first-order to zero-order clearance by the liver [6]. Overall, this results in a prolonged half-life of up to 30 hours for amlodipine, which is in stark contrast to the half-life of 1 to 2 hours for first-generation CCBs [5]. When tailoring a treatment plan, it is important to consider the wide variability in drug clearance and extent of toxicity in each individual patient based on exposure level and liver function.

Our patient ingested 150 mg of amlodipine, and arrived at our ED within 3.5 hours of ingestion. She received appropriate decontamination therapy with 50 g of activated charcoal after establishment of a definitive airway via endotracheal intubation. She exhibited cardiovascular collapse upon arrival to our ED, which manifested the severity of her exposure to CCB. Because of the dihydropyridine toxicity, she was hypotensive with a normal to marginally elevated heart rate, which was not an appropriate compensation for her shock state. Throughout her hospitalization, she did not require treatment of bradycardia or myocardial dysfunction. Standard treatment was provided upon arrival at our ED including intravenously administered crystalloid fluids, intravenously administered calcium gluconate, intravenously administered glucagon, intravenously administered insulin, and intravenously administered dextrose all in bolus form. Intravenously administered glucagon is controversial in CCB overdose, while it is well established for the treatment of beta-blocker overdose. This is due to its mechanism of increasing cyclic adenosine monophosphate (cAMP) and contraction of smooth muscle via a beta-receptor independent pathway [7]. It is, therefore, of questionable usefulness in CCB overdose mechanistically [4].

Hyperinsulinemia-euglycemia therapy has been utilized in the setting of CCB and beta-blocker overdose. There is evidence that this therapy improves hemodynamics in human observational studies [8], human case series [9–12], and animal studies [13–16]. The mechanism of action is incompletely understood, but CCBs appear to disrupt fatty acid metabolism and create relative insulin resistance within the myocardium. This is complicated by the impaired ability of the pancreatic beta-islet cell to secrete insulin via exocytosis, which is dependent on calcium influx. The high-dose insulin infusion is believed to partially compensate for this insulin-resistant state, by promoting carbohydrate delivery to the myocardium [17, 18]. Our patient received an initial intravenously administered bolus of 70 units of regular insulin, along with 50 g of dextrose intravenously administered. This was followed by an infusion of regular insulin at 70 units/hour. The typical dosing is 1 unit/kg of regular insulin bolus with 0.5 g/kg dextrose push, followed by 0.5 to 1 unit/kg per hour of regular insulin [19]. Refractory hypokalemia and hypoglycemia added further complication to the treatment of our patient, and the decision was made to abandon hyperinsulinemic-euglycemia therapy. There was no appreciable hemodynamic consequence to the discontinuation of this therapy indicating no significant component of insulin resistance.

As described above, amlodipine toxicity would be expected to have profound peripheral vasodilatory effects, while relatively sparing myocardial contractility and chronotropy. This was confirmed using point-of-care transthoracic echocardiography. The parasternal views revealed a hyperdynamic left ventricle, with under-filling and almost complete obliteration of her left ventricular cavity on short-axis view. This provided reassurance that there was no cardiogenic component to her shock state, and that cardiovascular collapse through profound peripheral vasodilatation was the primary component. Our decision to begin aggressive intravenously administered crystalloid resuscitation using a Level 1 rapid infuser was primarily to avoid systemic hypothermia. In the setting of profound vasodilatation, our patient was already predisposed to heat loss and hypothermia.

Intravenously administered lipid emulsion has been used in the setting of CCB overdose with mixed results, and there remains a great deal of controversy regarding its benefit. While its efficacy in the setting of amlodipine toxicity is poorly elucidated, it is reasonable to administer as rescue therapy in the setting of cardiac arrest due to CCB toxicity or during refractory shock and cardiovascular collapse [20–22]. We administered 1.5 mL/kg of 20% lipid emulsion as an intravenously administered bolus, as described previously [20, 23, 24]. There have also been studies using higher doses (6.2 to 18.6 mL/kg), although these were animal models [19]. In our patient, the administration of lipid emulsion therapy resulted in impaired laboratory monitoring due to gross lipemia and hemolysis, without immediate improvement in the hemodynamics. Specifically, serum potassium and lactate, as well as international normalized ratio/partial thromboplastin time (INR/PTT) became unreliable. It is important to note that there is evidence for up to three repeated doses, especially during cardiac arrest [19], and so it is possible we under-dosed our patient and we also did not continue a lipid emulsion infusion following the initial bolus. However, our patient started to show a remarkable resolution of her cardiovascular shock state over the next 12 hours following administration of the lipid emulsion therapy. Of interest, there is evidence for delayed hemodynamic improvement following lipid emulsion therapy of 10 hours in one case report [21]. In our case, we do not believe lipid emulsion therapy, at the limited dose provided, played a significant role in her recovery, although true causality cannot be established definitively.

Our patient’s dramatic recovery from her vasodilatory shock due to amlodipine overdose was a combination of our initial aggressive resuscitation, reversal of the anuric state, and fluid removal by means of CRRT. All organ dysfunction resolved, and she was successfully extubated during her third day of hospitalization. It became apparent at that time that she was having significant distress due to loss of vision bilaterally. She was previously documented to have mild optic atrophy with visual acuity of 20/40 bilaterally upon ophthalmology assessment as an out-patient in December 2007. Considering she described mild red and green images and no ability to perceive objects, this was a significant change from her baseline. Neuroimaging via MRI of her head revealed subtle cortical infarcts in her right frontal and parietal lobes, but would not explain her apparent blindness. Cerebral infarcts, while rare, have been described secondary to CCB overdose in the literature [2, 25]. Magnetic resonance angiography of her head and neck did reveal acute infarction of her optic nerves bilaterally, as well as the possibility of CNS vasculitis due to irregularities of the M2 and M3 branches of both MCAs. The differential for these findings included primary vasculitis or secondary causes, including drug overdose. Historically, drug-induced CNS vasculitis has been seen in the setting of cocaine or methamphetamine overdose [26–28]. Of importance, our patient did not exhibit any signs and symptoms of systemic vasculitis. An alternative explanation could be due to profound vasoconstriction caused by the high doses of norepinephrine and epinephrine that were required to support her. Of interest, reversible cerebral vasoconstriction syndrome (RCVS) has been described previously in the setting of epinephrine administration [29]. RCVS is a relatively rare clinical and radiological syndrome encompassing a diverse group of disorders leading to reversible vasoconstriction of the cerebral arteries. This vasoconstriction can be spontaneous or exogenously driven. The use of vasoactive drugs accounts for approximately 50% of cases in most published series [30]. While the pathogenesis is largely unknown, dysregulation of cerebral vascular tone may be induced by sympathetic overactivity, endothelial dysfunction, and oxidative stress. This is further supported by the significant overlap between RCVS and posterior reversible encephalopathy syndrome (PRES) [30]. Of interest, our patient did have focal stenosis of an M2 branch of her right MCA, with accompanying irregularities of several other M2 and M3 branches identified on MRI within several days of her presentation. A follow-up study with MRA of her head and neck 17 days later revealed no further M2 stenosis and resolution of described vascular abnormalities. Given the generally benign course of RCVS and reversibility within 12 weeks of onset, it is possible an element of RCVS was present, although it would not specifically explain focal and bilateral optic nerve infarction in our patient.

Our literature search failed to identify any previous case reports or retrospective studies of outcome data that describe blindness in the setting of CCB overdose. In this case report, we describe the first incident of blindness in a patient with CCB overdose. Overall, the most likely explanation for this patient’s bilateral blindness was probably multifactorial. She had a predisposed condition of bilateral optic atrophy at baseline. This had certainly progressed, since her original assessment in 2007. She was in a profound state of shock upon admission, and this could theoretically have caused significant hypoperfusion to smaller vessels supplying the optic nerves. Blindness secondary to systemic hypotension has been described previously in the literature, and is often due to profound or recurrent blood loss [31]. In general, culprit lesions are located within the parietal or occipital lobes in the setting of systemic hypotension, by means of infarctions in the border zones between cerebral arteries, or due to diffuse cerebral cortical neuronal death [31, 32]. Other mechanisms for visual loss after hypotension include juxtalaminar optic nerve infarction in the setting of atherosclerosis, and orbital optic nerve infarction due to compression from hypoxic edema [31, 32]. When optic nerve infarcts are present without the finding of watershed infarcts, a secondary underlying factor must be present to cause vulnerability within the optic nerves. Other case reports have identified localized atherosclerosis or severe anemia as a secondary risk factor. In our patient, the pre-existing optic nerve atrophy led to vulnerability of the optic nerves for infarction in the setting of systemic hypotension.

An important condition to consider is anterior ischemic optic neuropathy (AION), which is characterized by a sudden loss of vision due to optic nerve head ischemia. Infarction of the short posterior ciliary artery supplying the optic nerve head is believed to be the culprit, causing ischemia, edema, and compartment syndrome [33]. AION is subdivided into arteritic (10%) and non-arteritic (90%) causes [34]. Non-arteritic AION (NAAION) occurs in the setting of compromised blood flow associated with several comorbidities including hypertension, tobacco smoking, hypercholesterolemia, atherosclerosis, and diabetes mellitus [33, 34]. An anatomically small or “crowded” disc has also been identified as a risk factor [33]. The clinical presentation includes painless visual loss and swelling of the optic disc, followed by pallor. NAAION can present with visual loss upon waking, due to nocturnal hypotension [33]. NAAION typically involves unilateral visual loss, but 14.7% of patients over a 5-year follow-up period in the ischemic optic neuropathy (ION) decompression trial progressed to second eye involvement [35]. In most cases of hypotension-induced NAAION, bilateral visual loss occurs simultaneously or within weeks of the initial unilateral symptoms [33]. NAAION has also been described in patients undergoing chronic renal replacement therapy, but is largely underdiagnosed in this population [33, 34, 36]. Of interest, not only patients undergoing intermittent hemodialysis are at risk. In a case report by Al-Kaabi *et al*., a 5-year-old child undergoing continuous peritoneal dialysis (CPD) for a period of 4 years developed acute bilateral vision loss upon waking from sleep [34]. In this patient, frequent systemic hypotensive episodes and one instance of PRES had required previous hospitalization. A funduscopic examination was consistent with NAAION, and anemia, uremia, as well as infiltrative optic neuropathy and other intracranial causes were ruled out. The resulting diagnosis was NAAION caused by hypotension-induced low perfusion and ischemia of the optic nerve [34]. Vidal and Schaefer reviewed the occurrence of AION in infants, and found it to occur in 1% of children on CPD [36]. Risk factors in this population included very young age, autosomal recessive polycystic kidney disease, and sustained hypotension [36]. While poor visual outcomes have been reported among both children and adults undergoing CPD diagnosed with NAAION, the primary treatment remains reversal of hypotension and optimization of optic nerve perfusion [34]. Steroid therapy has also been shown to provide some therapeutic effect, although it remains controversial [37–39].

NAAION due to hypotensive insult is not isolated to those undergoing chronic renal replacement therapy. Kim *et al*. published a case report on a 50-year-old man following a lumbar laminectomy, with subsequent prolonged immobilization for a 3-month period [40]. Upon sitting upright for the first time since his operation, he experienced significant orthostatic hypotension. Within several hours, he developed acute visual loss and was ultimately diagnosed with NAAION by means of optic disc filling delay on fluorescein angiography [40]. Of interest, he was also found to be anemic, a known secondary risk factor for the development of NAAION [33]. Partial recovery of vision was noted in this patient upon 6-month follow-up, although the visual fields remained severely constricted [40].

Perioperative vision loss (POVL) following prone spine surgery has been described in the literature, occurring in 0.013 to 1% of cases [41]. While AION has been identified in some cases, other causes include posterior ischemic optic neuropathy (PION), central retinal artery occlusion, central retinal vein occlusion, cortical blindness, direct compression, acute angle closure glaucoma, epidural spine injections, and other less common factors [41]. In terms of AION in this setting, identified risk factors include prolonged operative times, long-segment spinal instrumentation, anemia, intraoperative hypotension, diabetes, obesity, male sex, greater estimated blood loss, microvascular pathology, and decreased percent colloid administration [41].

Hypotension-induced NAAION can also occur following acute blood loss and in patients prescribed medications such as phosphodiesterase type 5 inhibitors and interferon-α [34, 42, 43]. In terms of hemorrhagic shock, NAAION can result from both spontaneous and traumatic blood loss [31, 40, 44–46]. Reported outcomes have ranged from some degree of vision recovery in 50% of patients, while 10 to 15% of patients experienced complete recovery of vision [40]. Overall, hypotension-induced NAAION could help explain the pathophysiology of bilateral vision loss in our patient, although a definitive diagnosis would have required further diagnostic testing in the setting of chronic optic atrophy.

The high doses of vasopressors required in this case may have had more pronounced local effects in some smaller vessels, which could render structures like the optic nerves more vulnerable to infarction. The description above of RCVS does support cerebral vasoconstriction in the setting of vasoactive medications, although MRI revealed unilateral reversible stenosis of only the right MCA branches in our patient. While direct ischemia of the optic nerves has not previously been described in the setting of CCB overdose, ischemia within other structures is well described in the literature. Above, we described case reports of cerebral infarction following CCB overdose. Other case reports and retrospective analyses have described bowel ischemia and renal ischemia due to high-dose vasopressor use in the setting of CCB overdose [47, 48].

Our patient was seen in follow-up by her ophthalmologist as an out-patient 5 months’ post-discharge. Unfortunately, her vision measured no light perception bilaterally without pupillary reaction to light. She was diagnosed as having complete optic atrophy suggestive of severe optic nerve damage which was felt to be irreversible.

## Conclusion

In the setting of a CCB overdose causing persistent severe hypotension secondary to profound vasodilatory shock, multiple high-dose vasopressors support for the cardiovascular collapse can lead to worsening of existing optic atrophy resulting in bilateral blindness.

## Abbreviations

ACA: Anterior cerebral artery
AION: Anterior ischemic optic neuropathy
ATN: Acute tubular necrosis
cAMP: Cyclic adenosine monophosphate
CCB: Calcium channel blocker
CNS: Central nervous system
CPD: Continuous peritoneal dialysis
CRRT: Continuous renal replacement therapy
D50W: Dextrose 50% in water
DWI: Diffusion-weighted image
ECG: Electrocardiogram
ECMO: Extracorporeal membrane oxygenation
ED: Emergency department
FiO_2_: Fraction of inspired oxygen
FLAIR: Fluid-attenuated inversion recovery
GCS: Glasgow Coma Scale
INR/PTT: International normalized ratio/partial thromboplastin time
ION: Ischemic optic neuropathy
MCA: Middle cerebral artery
MRA: Magnetic resonance angiography
MRI: Magnetic resonance imaging
NAAION: Non-arteritic anterior ischemic optic neuropathy
PEEP: Positive end-expiratory pressure
PION: Posterior ischemic optic neuropathy
POVL: Perioperative vision loss
PRES: Posterior reversible encephalopathy syndrome
RCVS: Reversible cerebral vasoconstriction syndrome
RV: Right ventricle
